# Improving the SERS signals of biomolecules using a stacked biochip containing Fe_2_O_3_/Au nanoparticles and a DC magnetic field

**DOI:** 10.1038/s41598-019-45879-5

**Published:** 2019-07-02

**Authors:** Zu-Yin Deng, Kuen-Lin Chen, Chiu-Hsien Wu

**Affiliations:** 1 Department of Physics, National Chung Hsing University, Taichung, 402 Taiwan; 2 Institute of Nanoscience, National Chung Hsing University, Taichung, 402 Taiwan

**Keywords:** Biosensors, Nanophotonics and plasmonics

## Abstract

This study proposes a magnetic biochip that uses surface-enhanced Raman scattering (SERS) for antigen detection. The biochip was a sandwich structure containing alternating layers of gold and magnetic Fe_2_O_3_ nanoparticles. Both single (Au/Fe_2_O_3_/Au) and multilayer (Au/Fe_2_O_3_/Au/Fe_2_O_3_/Au) chips containing Fe_2_O_3_ nanoparticles were fabricated to detect bovine serum albumin (BSA). The single-layer chip detected the BSA antigen at a signal-to-noise ratio (SNR) of 5.0. Peaks detected between 1000 and 1500 cm^−1^ corresponded to various carbon chains. With more Fe_2_O_3_ layers, bond resonance was enhanced via the Hall effect. The distribution of electromagnetic field enhancement was determined via SERS. The signal from the single-layer chip containing Au nanoparticles was measured in an external magnetic field. Maximum signal strength was recorded in a field strength of 12.5 gauss. We observed peaks due to other carbon–hydrogen molecules in a 62.5-gauss field. The magnetic field could improve the resolution and selectivity of sample observations.

## Introduction

Raman spectroscopy, a fast, non-contact, and non-destructive optical method, can be used to real-time observation of molecular structures at room temperature. Thus, this method can be used for continuous measurements of biological reactions. However, most biomolecular materials show no obvious peaks in Raman spectra because they contain folded hydrocarbon chains bound together by strong interactions. The intermolecular force broadens the signals and produces wave packets in the Raman spectrum^1–3^.

Surface-enhanced Raman scattering (SERS) has been extensively used to enhance the signals from biomolecules^4–7^. The laser power of the Raman microscope excites the electrons in precious-metal particles, producing surface plasmon resonances (SPRs). The local electric field in SPR releases electrons and influences the bond vibrations. Certain binding energies form shapes with clear peaks in the Raman spectrum. This method provides a highly sensitive, label-free bioassay, in which the specific antigen type can be immediately distinguished^6,7^.

The SERS enhancement factor is represented as follows^8^:

1 $${G}_{SERS}({r}_{m},v)={|\frac{E({r}_{m},v)}{{E}_{inc}(v)}|}^{4}$$

Here, *E*(*r*_*m*_, *v*) is the total electric field where *r*_*m*_ is the distance from the metallic particle to the molecule, *v* is the laser frequency, and *E*_*inc*_ is the electric field excited by incident electromagnetic waves. Stronger signals can be obtained from biological samples by the enhanced electromagnetic field of a SPR.

The SERS signal strength is directly determined by the power and wavelength of the excitation laser. However, the high energy destructs the biomolecules. Metallic nanoparticles have been previously used to improve and optimize the SERS signals from biomolecules. Gold and silver nanoparticles were fabricated into tips of different shapes and surface areas. Other approaches, such as core-shell and hollow structures as well as material mixing, have been employed to increase the electric field of SPR^9–13^. Herein, magnetic nanoparticles are used to label biomolecules in many bioassay methods using Raman spectroscopy. Biosamples were connected to magnetic nanoparticles, which were grouped in an external magnetic field. The groups, including target biomolecules in agents, were deposited on the Raman-detected interface. This process provides a selection of biomolecules and enables the detection of larger numbers of molecules and the measurement of strong signals^3,14^.

Herein, magnetic fields are used to enhance the SPR and Raman signal from an antigen. Biochip stacks with different layers of Fe_2_O_3_ nanoparticles are fabricated on an SiO_2_ wafer^15,16^. An external magnetic field generated by a coil is used to determine the magnetic effect on the SERS. The biochip and magnetic field are optimized for BSA detection^17^.

## Results

### Magnetic SERS biochip

**Figure 1:**
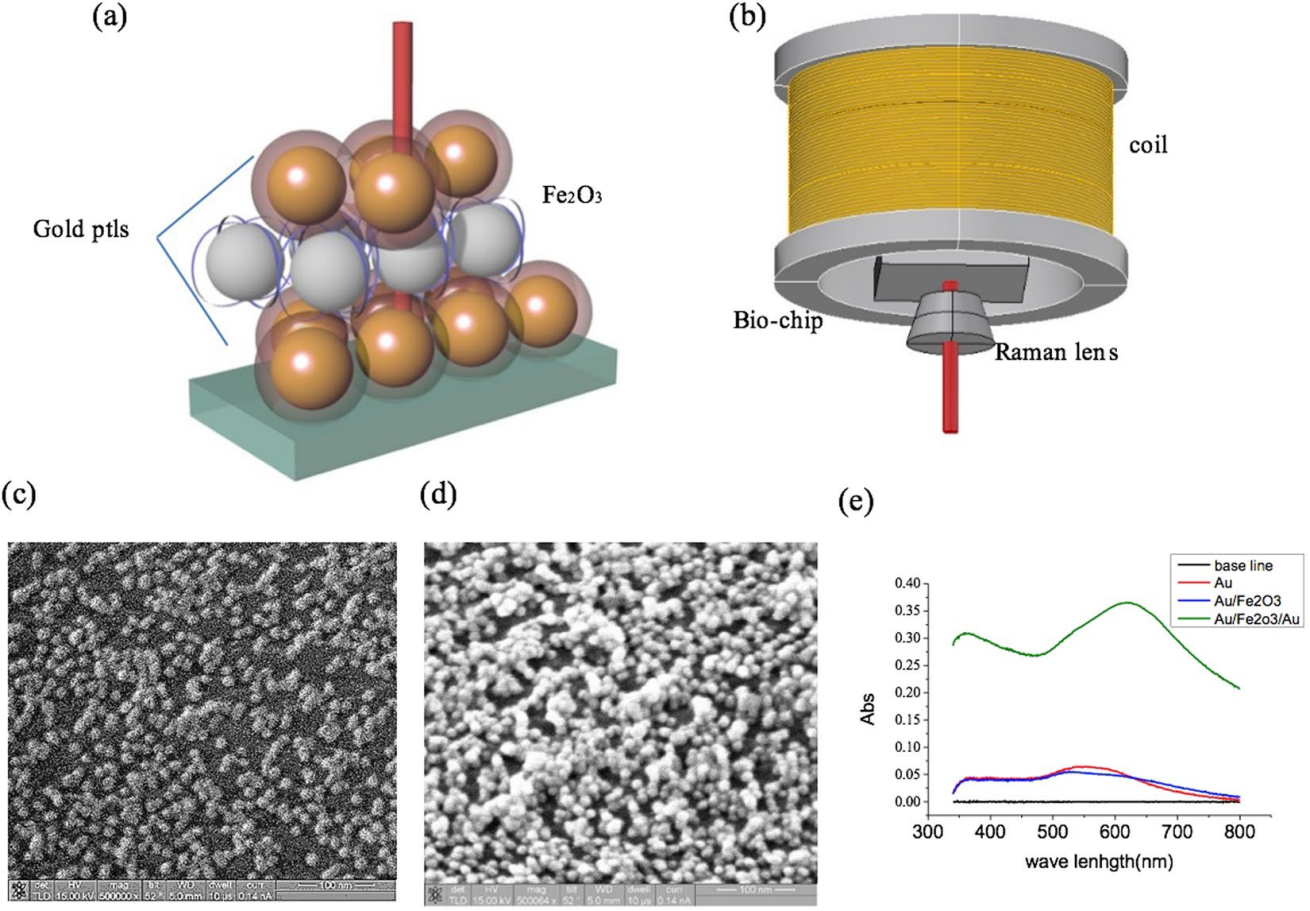
Architecture of system diagram. (**a**) Particle based biochip. (**b**) Biochip side in Raman system with magnetic coil set. (**c**) SEM image of gold particles coated on SiO_2_ wafer. (**d**) Single-layer iron-oxide ptl film (Au/Fe_2_O_3_/Au). (**e**) Absorption of chip with stacked ptls.

**Figure 2:**
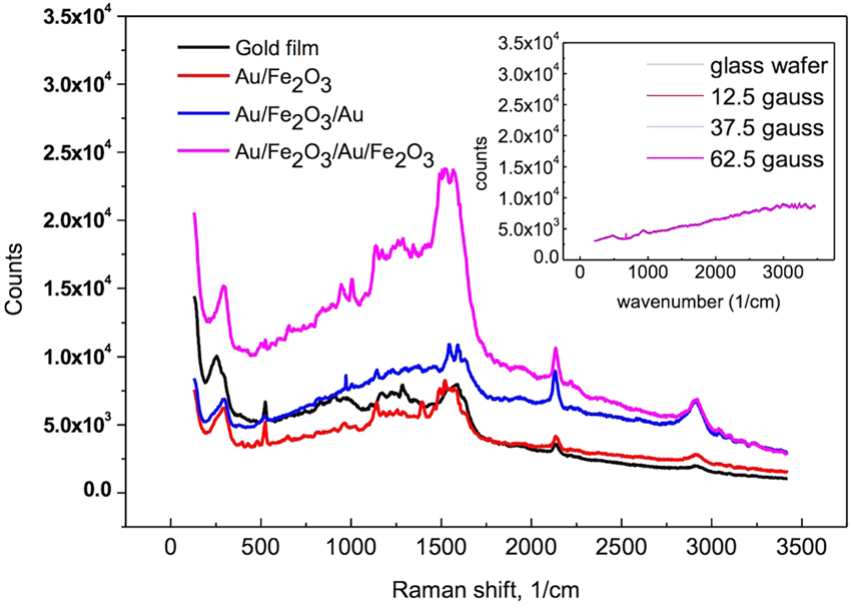
Raman spectra of difference layer of bio-chip Illustration shows the signal of the glass substrate. The laser intensity is 0.6 mW and the acquisition time is 30 sec.

Before coating a biochip with antibodies, we imaged its surface morphology and binding structure using a scanning electron microscope. We first fixed a single layer of gold nanoparticles on an SiO_2_ wafer using (3-aminopropyl)-trimethoxysilane (APTMS) [Fig. 1(a–c)]. We then gradually attached successive layers of iron-oxide and gold nanoparticles to form Au/Fe_2_O_3_/Au layered structures (Fig. 1d). We determined the thickness of the nanoparticle film via UV–VIS spectroscopy. The absorption peak strengthened and shifted to longer wavelengths with the addition of more nanoparticle layers (Fig. 1e). Thus, the biochip absorbed maximum Raman laser (633 nm) radiation, indicating the SPR enhancement in the gold nanoparticles. Raman signals from the binding functional groups of multilayer chips are shown in Fig. 2. The chip with a single layer of gold nanoparticles showed peaks in the four spectral regions 400–600, 1000–1700, 2700, and 2900 cm^−1^ (Fig. 2). Comparison with a database of Raman spectra shows that the signals from the biochip correspond to various carbon-containing organic molecules^18^. The peak at 442.9 cm^−1^ represented the S–S single bond, whereas the region from 1000to 1700 cm^−1^ corresponded to the C–C, C=S, and CH_2_ bonds. Gold nanoparticles produced no obvious peaks, and the peak due to iron oxide was near 100 cm^−1^. The background signal from the SiO_2_ wafer did not produce peaks, as shown in the inset of Fig. 2. These signals also did not change with the external magnetic field of 0–62.5 gauss.

We used the signal-to-noise ratio (SNR) to describe the signal improvement. The signal intensity was determined by counts (*Cnts*) on the CCD. The background, *Cnts*_*Bg*_, was chosen as the counts at 3500 cm^−1^ to avoid the protein background wave package. The SNR is defined as

2 $$SNR=\frac{Cnt{s}_{Peak}}{Cnt{s}_{Bg}},$$

where *Cnts*_*peak*_ is the count at the peaks.

The signals of the double-layer (Au/Fe_2_O_3_/Au/Fe_2_O_3_) chip was 4–5 times greater than those of the chip containing only single layer (Au/Fe_2_O_3_), as shown in Fig. 2. Thus, a magnetic field effect on the SERS is observed. The magnetic beads had weak magnetic fields, which passed through the entire layer of gold nanoparticles on the biochip^18^. The magnetic field combined with the electromagnetic field on the particle surface according to the Lorentz force law:

3 $$\mathop{{\rm{F}}}\limits^{\rightharpoonup }={\rm{q}}(\mathop{{\rm{E}}}\limits^{\rightharpoonup }+\mathop{{\rm{v}}}\limits^{\rightharpoonup }\times \mathop{{\rm{B}}}\limits^{\rightharpoonup }),$$

where F is the force acting on the electrons, E and B are the electric and magnetic fields, respectively, and q and v are the electric charge and the velocity of the electrons, respectively. Free electrons on the surface of a gold nanoparticle are polarized by this force. We combined this polarizing electric field into the field of the SPR as follows:

4 $$E={E}_{Maxwell}+{E}_{S}+{E}_{near}.$$

In other words, the total electric field *E* of the SPR comprised the electromagnetic field *E*_*Maxwell*_, the polarization field *E*_*s*_, and a field due to dipoles *E*_*near*_^19–23^. The enhanced polarization caused an electron overlay to the SPR of the gold nanoparticles. The SPR was enhanced by increasing the amount of Fe_2_O_3_ nanoparticle layers because of the higher electric-field polarization.

### Bioagent enhancement of the Raman signal

**Figure 3:**
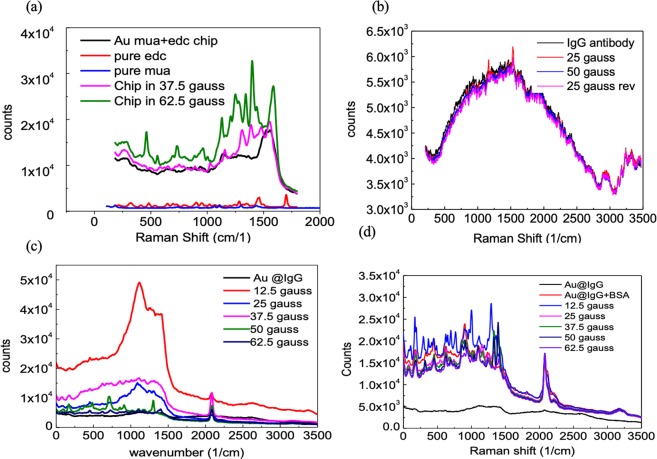
Protein sample measurement. (**a**) Gold particle film surface was treated with functional carbon chain. (**b**) IgG antibody agent paved on glass wafer, (**c**) IgG antibody was coated on gold particle film bio-chip. (**d**) After immune response with BSA. The laser intensity is 0.6 mW and the acquisition time is 30 sec.

We measured a biochip containing a single layer of gold nanoparticles in an external magnetic field [Fig. 3(a)] and found that a signal peak appeared at 1500 cm^−1^. This figure also shows are peaks due to C(NO_2_) (1300–1400 cm^−1^) and CH_2_ (1425 cm^−1^). We detected peaks due to the S–S, C–S, O–O, and C–C bonds (1000–1300 cm^−1^) in the stronger field of 62.5 gauss. We measured the antibody immunoglobulin G (IgG) on a SiO_2_ wafer for specific immune reactions to BSA before coating it onto the chip [Fig. 3(b)]. This signal appears as a wave packet between 900 and 1500 cm^−1^, and there was no obvious change in the signal with magnetic field strength.

For specific antigen detection, we coated the antibody IgG on the biochip as the immune reagent. We then measured the biochip in an external magnetic field, as shown in Fig. 3(c). The signal exhibited a wave package between 750 and 1500 cm^−1^, and the signal considerably decreased after the antibody was covered onto the chip. The maximum signal appeared in a field of strength 12.5 gauss. We measured a sharp peak at 1100 cm^−1^ and two shoulders between 1300 and 1500 cm^−1^. SNR was enhanced to 8.7–9.2 (Table 1). As the electromagnetic field strength increased to 37.5 gauss, the peaks formed due to C–C and C–S decayed as a wave packet. In a field of 50 gauss, the signal due to IgG decayed to the original levels obtained before the application of the magnetic field. Simultaneously, peaks were enhanced between 400 and 1000 cm^−1^. The positions of these peaks correspond to the signal of empty biochip in the field of 62.5 gauss. We consider these signals to reflect the chemicals used in the processing due to the structural differences between IgG and the other functional organic groups. These long-carbon-chain molecules are composed of covalent bonds. However, the winding and folding of 3D protein structures are based on many weak bond interactions, which are easily affected by the additional electric fields associated with the SPR. Thus, the strength of the Raman signal obtained from proteins decreased in higher magnetic fields.

**Table 1:** Peaks of IgG antibody coated on gold film.

Functional group	Region (cm^−1^)	Origen SNR ratio	Enhanced SNR ratio
CC aliphatic	359.6	n/a	4.8
S–S	442.8	n/a	4.4
C–S	617	3.1	5.4
CC bond	1116	3.8	11.1
C=S	1248	3.7	9.2
C–(NO_2_)	1340–1400	3.7	8.7
C=C	1600–1700	n/a	2.49–2.98
C=O	1700–1850	n/a	2.44–2.61
C≅C	2079	2.9	2.6

We placed a drop of BSA antigen on the biochip for the immune test. Figure 3d shows a plot of the results after we immunized the target antigen BSA with the IgG antibody. We found peaks distributed at 300–1500 cm^−1^, corresponding to the C–C aliphatic, C–S, and O–O bonds. The best enhancement occurred in a 12.5-gauss field. A comprehensive database can be established by exciting a larger number of peaks (Table 2). In this experiment, the signals due to the antibody and the antigen exhibited main peaks were magnified eight times by the 12.5-gauss external field. At higher field strengths, the protein signals decreased and the biochip signal was detected in field strengths over 50 gausses. This result was similar to that obtained from the IgG antibody. Selectivity of the protein measurement was provided by the external magnetic field, enabling us to distinguish different bonds.

**Table 2:** SERS signals of BSA antigen immune react on gold film.

Functional group	Region (cm^−1^)	Origen SNR ratio	Enhanced SNR ratio
CC aliphatic	359.6	n/a	7.3
S–S*	442.8	7.8	7.3
C–S*	622	7.4	8.5
O–O*	905	9.0	9.0
CC bond	1116.536	7.0	10.7
C=S	1236.089	6.9	11.2
C–(NO_2_)	1340–1400	n/a	8.4
CH_2_	1425	n/a	7.3
C≅C	2079.02	3.1	6.1
C–H*	3184	1.8	1.8

*Signals appeared after IgG immune with BSA.

### Magnetism biochip measurement

**Figure 4:**
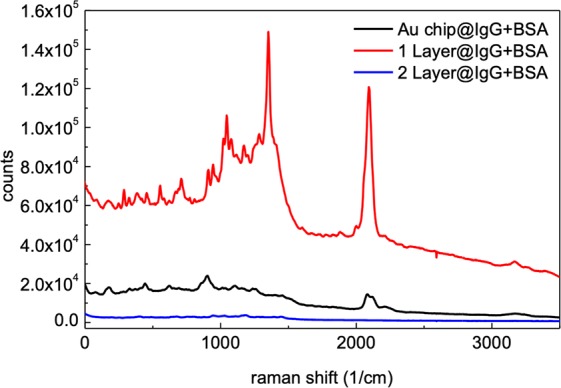
BSA antigen immune on single (Au/Fe_2_O_3_/Au) and double (Au/Fe_2_O_3_/Au/Fe_2_O_3_/Au) irion-oxide layer biochip. The laser intensity is 0.6 mW and the acquisition time is 30 sec.

The density of the magnetic field increased with increasing magnetic nanoparticle layers. We fabricated biochips with both a single (Au/Fe_2_O_3_/Au) and a double (Au/Fe_2_O_3_/Au/Fe_2_O_3_/Au) layer of iron oxide. The SPR was enhanced by the magnetic nanoparticles, similar to the enhancement produced by the external magnetic field from the coil. The upper gold layer bonded with the protein molecules. The signal of the BSA immune response is shown in Fig. 4. The largest increase in the spectrum signal occurred for the single-layer, magnetic-bead biochip, which enhanced the spectral range within 800–1500 cm^−1^. We also detected sharp peaks at 1116 cm^−1^ (C–C), 1400 cm^−1^ (C–NO_2_), and 2100 cm^−1^ (C≅C). The peak height was 10 times larger than the BSA immune response from the chip with the single layer of gold nanoparticles. However, the signals decreased for the chip with the double layer of Fe_2_O_3_ nanoparticles. Compared with the result of the extra-field experiment, more peaks were enhanced by the magnetism of the beads. The signal reflected as wave packets between 800 and 1500 cm^−1^. The magnetic beads were located close to the gold nanoparticles and protein molecules, thus providing a more-uniform field and stronger interactions. The Raman signal due to the proteins decreased in the stronger magnetic fields, both in the double-bead layers and in external fields over 37.5 gauss. These results indicate that the signal due to BSA was amplified by a suitable magnetic field. The protein structure was affected by the extremely strong electric field caused by the Hall effect. The peak counts and sensitivity of the BSA antigen on a single-layer iron-oxide chip are listed in Table 3. Compared to the chip with gold nanoparticles only, the single-layer iron-oxide chip showed enhanced peaks between 1200 and 2100 cm^−1^. Most double carbon bonds and the C–(NO_2_) bond were measured as sharp peaks, and the sensitivity was enhanced 10–30 times.

**Table 3:** Peaks of BSA antigen immune react on the chip single layer Fe_2_O_3_.

Functional group	Region (cm^−1^)	Chip with IgG	IgG + BSA
CC aliphatic	359.6	8.0	2.2^a^
S–S	442.8	8.3	2.2
CC bond	1116	10.7	3.5
C=S	1248	n/a	3.0
C–(NO_2_)	1340–1400	9.6	5.0^a^
C=C	1600–1700	n/a	1.6
C=O	1700–1850	n/a	1.5
C≅C	2079	5.8	4.0

^a^Signals of BSA antigen didn’t showed in the measured with gold ptls chip.

**Figure 5:**
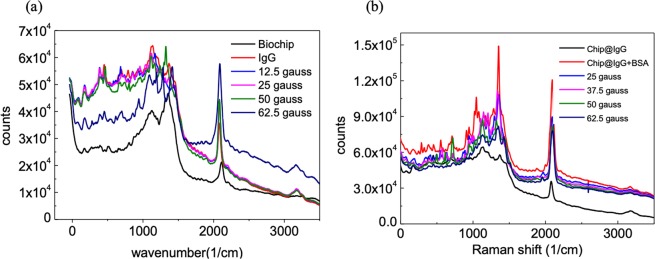
Raman signal of proteins coated on single layer iron-oxide bio-chip. (**a**) IgG antibody. (**b**) Immune with BSA antigen The laser intensity is 0.6 mW and the acquisition time is 30 sec.

To determine the best measurement conditions, we added an external field to influence the single-iron-oxide-layer chip using external fields from 0 to 62.5 gauss. Figure 5a shows the Raman signal of the antibody IgG. Before the field was added, we observed two peaks between 900 and 1500 cm^−1^. We found that the signal from the IgG enhanced these two peaks and that the intensity at 1200 cm^−1^ was greater than that at 1300 cm^−1^. We also detected other signals at 0–1000, 2100, and 3200 cm^−1^, and the intensity of iron-oxide chip was 10 times stronger than that from the gold chip. The signals continued to decrease with increasing strength of the external magnetic field. At 62.5 gauss, signals similar to that of the biochip were observed. Figure 5b shows the signals with the BSA reagent covered up the chip with IgG. The signals decreased in a higher external field.

After being subjected to the magnetic field, the signals did not return to their original levels because the protein structure underwent no observable changes in the magnetic field, as shown in Fig. 3b. Therefore, we presume that the protein structure may have been affected by the excess electric field on the metal surface. The biomolecular resonance was enhanced by the magnetically enhanced SPR. The SERS was also enhanced by the magnetic field below the critical point for molecular structural change. We achieved the best signals from the chip with the single layer of magnetic beads. The positions of the peaks between and 1500 cm^−1^ showed the most significant enhancements and changes in shape. In contrast, the proteins and functional molecules for fixed nanoparticles had a different response in the magnetic field. We expected the external field to enable us to achieve high selectivity and high resolution in biosensing.

**Figure 6:**
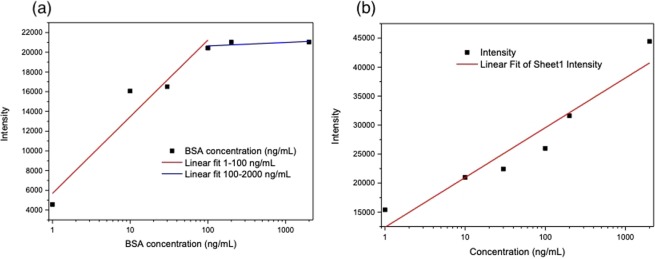
The SERS intensity of BSA concentration 1–2000 ng/mL immune with IgG (**a**) gold npls chip with external magnetic field 12.5 gauss. (**b**) Single (Au/Fe_2_O_3_/Au) irion-oxide layer biochip. The laser intensity is 0.6 mW and the acquisition time is 10 sec.

For the gold npl chip, the peak intensity of C–S bond (622 cm^−1^) is highly sensitive to the magnetic field. And hence it was used to evaluate the dependence of BSA concentration. As shown in the Fig. 6a, the peak intensity of C–S bond is monotonically increased with the BSA concentration during 1~100 ng/mL and approaches to equilibrium when the BSA concentration was larger than 100 ng/mL. On the other hand, for the chip with single layer of iron oxide(Au/Fe2O_3_/Au), the signal of C–(NO_2_) bond (1300–1400 cm^−1^) is used to evaluate the dependence of BSA concentration. Figure 6b shows that the peak intensity of C–(NO_2_) bond is also monotonically increased with the BSA concentration in the range between 1~2000 ng/mL. Moreover, this method was used to analyze different kinds of proteins. Based on the experimental data, the SERS signals enhancement was observed in the sample of hemocyanin as shown in the Fig. S5. However, the SERS enhancement effect was not observed in the samples of C-reactive protein (CRP) and Biotin. We inferred that the intensity of Raman resonance depends on the sample absorption of light and the distance between molecules and metal particles^24^. The external could enhanced the SPR of bio sample without change the binding structure. Therefore, molecules with SERS signals could be improved by this method.

## Discussion

We used appropriate magnetic field intensities to enhance the Raman signals of biomolecules. We excited the molecular vibrations using a magnetic chip and an external magnetic field generated by a coil. This resulted in the separation and enlargement of more peaks than was the case for reactions on a chip containing only gold nanoparticles. The resolution the BSA antigen was enhanced to 3–5 when we used the chip with a single iron-oxide layer. Protein structures considerably differ from other chain-shaped carbon-based molecules. The molecules for binding antibody were excited in a 62.5-gauss field higher than the field of the reacting protein. We conclude that the weak binding energy for protein folding was changed by the SPR electric field. By enhancing the specific protein signal, standard maps could be established. Thus, this method can be widely used to measure the material quality and content ratios. In biological detection, protein structures can be observed directly without requiring any irreversible chemical treatment. Samples can be repeatedly measured, and their evolution can be recorded over time. These advantages make this magnetic biochip a powerful tool for biological tests. Our future study will focus on the cross interference between different species of protein, re-usability of substrates, and the overall sensitivity of the device.

## Methods

We fabricated a biochip for specific immune detection. The required materials and chemicals included (3-aminopropyl)-trimethoxysilane (APTMS) 95%, 11-mercaptoundecanoic acid (11-MUA), 1-ethyl-3-[3-(dimethyl amino) propyl] carbodiimide (EDAC), immunoglobulin G (IgG), bovine serum albumin (BSA) acquired from Sigma-Aldrich, and 97% N-hydroxysuccinimide (NHS), ethylene glycol, chloroauric acid, and n-octylamine were from Alfa. Ferric chloride (FeCl_3_) was from ECHO.

We developed the biochip as an alternating sandwich structure of gold and Fe_2_O_3_ nanoparticles on a glass wafer. The gold particles were synthesized using chloroauric acid via aqueous citrate reduction^15^. The iron-oxide particle was synthesized via hydrolysis^16^.We immersed the cleaned wafer in a 1% APTMS–MeOH solution for 1 h to coat it with free amino groups. We then washed the wafer in deionized water three times and placed it in a solution of Au nanoparticles for 1.5 h. We placed the film of gold nanoparticles into a solution of APTMS-coated Fe_2_O_3_ nanoparticles for 1 h. We repeated these steps to build up the sandwich structure of gold and Fe_2_O_3_nanoparticles. Chips were rinsed with water and MeOH to remove any unbonded nanoparticles. The stacking of the nanoparticles is shown in Fig. 1a.

To detect a specific antigen, we covered the biochip with a layer of antibody on the surface. We immersed the nanoparticle film in 20 mM of an 11-MUA–MeOH solution for 16 h. After rinsing with water, we immersed the chip in EDAC (150 mM) and NHS (30 mM) for 30 min and placed a drop of antibody IgG PBS reagent (8 *μM*) on the chip^23^. After reacting and fixing the functional groups of EDAC and NHS, this chip exhibited specific immunity to BSA agents (8 *μM*).

We used a Raman microscope (Tokyo Instruments, Nanofinder 30) for the measurements reported herein. We set the wavelength and intensity of the exciting laser as 633 nm and 0.6 mW, respectively. The resolution of x-, y-, and z-axis were 320 nm and the acquisition time was 30 s. The number of grating was 300. To add a magnetic field generated by a coil, as shown in Fig. 1b, we oriented the magnetic field perpendicular to the sample.

## Supplementary information

SUPPLEMENTARY INFO
