# Supplementary material for: Improving the SERS signals of biomolecules using a stacked biochip containing Fe2O3/Au nanoparticles and a DC magnetic field
Source: Sci Rep. 2019 Jul 2;9:9566. doi: 10.1038/s41598-019-45879-5 (PMC6606591; doi:10.1038/s41598-019-45879-5)
Supplement: Supplementary file 1 — SUPPLEMENTARY INFO [file 41598_2019_45879_MOESM1_ESM.docx]

**Improving the SERS signals of biomolecules by using a stacked biochip containing Fe2O3/Au nanoparticles and a DC magnetic field**

Zu-Yin Deng1, Kuen-Lin Chen 1*, Chiu-Hsien Wu1,2*

**1Institute of Nanoscience, National Chung Hsing University, Taichung 402, Taiwan 2Department of Physics, National Chung Hsing University, Taichung 402, Taiwan *Corresponding author’s e-**mail:chwu@phys.nchu.edu.tw , [klchen@phys.nchu.edu.tw](mailto:klchen@phys.nchu.edu.tw)


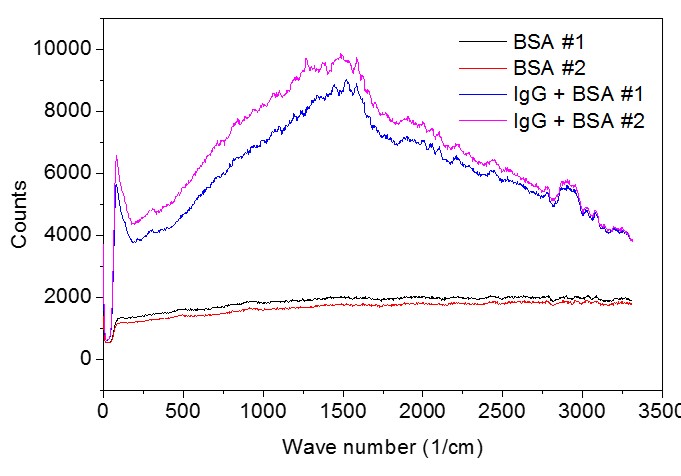


Figure S1. Raman signal of pure BSA regent and IgG immune react with BSA.


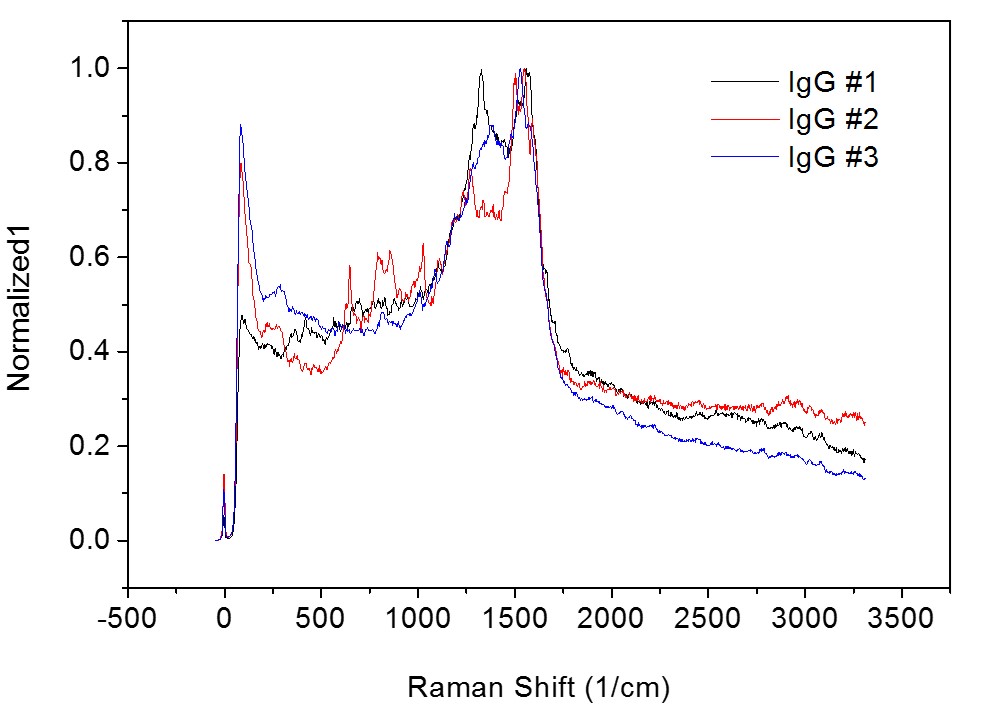


Figure S2. SERS spectrum repeatability of bio-chip with Antibody igG .


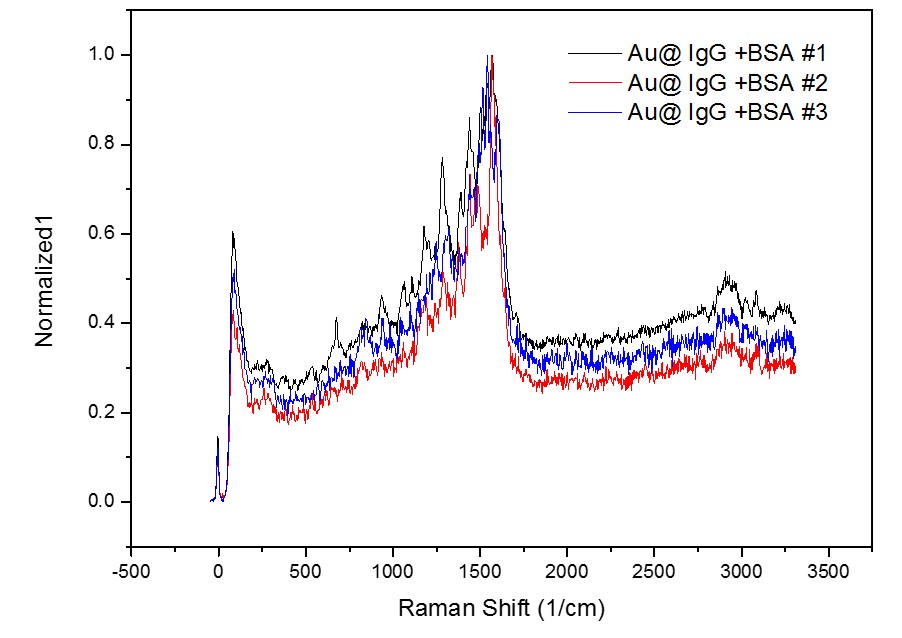


Figure S3. SERS spectrum repeatability of bio-chip with Antibody igG immune react with BSA .


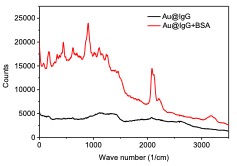


Figure S4. SERS spectrum of bio-chip with Antibody IgG before and after immune react with BSA .

Figure S5. SERS spectrum repeatability of bio-chip with Hemoglobin.

The laser power was 0.6mW, for 30s. External magnetic field was 12.5 – 37.5 gauss.
